# A critical role of farnesol in the modulation of Amphotericin B and Aureobasidin A antifungal drug susceptibility

**DOI:** 10.1080/21501203.2022.2138599

**Published:** 2022-10-28

**Authors:** Venkatramanan Mahendrarajan, Vinay Kumar Bari

**Affiliations:** Department of Biochemistry, School of Basic Sciences, Central University of Punjab, VPO-Ghudda, Bathinda, India

**Keywords:** *Candida*, Farnesol, Sphingolipid biosynthesis, Amphotericin B, Aureobasidin A

## Abstract

*Candida albicans* and its related species can cause opportunistic infections such as “candidiasis” in immunocompromised individuals with a high morbidity and mortality rate. Several antifungal drugs available in the market are often used to treat infections caused by pathogenic fungi. However, in fungi, the development of resistance against these drugs quickly evolved. *Candida* is a dimorphic fungus that can switch between yeast to hyphae form, requires an active biosynthesis of membrane constituents. Sphingolipid and ergosterol molecules, are the major fungal plasma membrane components, and their interaction with the antifungal drug can modulate drug susceptibility. A lipophilic compound farnesol acts as a quorum-sensing molecule synthesised by the isoprenoid biosynthesis pathway in the fungal pathogen *Candida*. Farnesol is secreted in a cell density-dependent manner inhibits hyphae germination and biofilm formation. In this study, we have investigated whether the farnesol molecules affect the drug susceptibility of the antifungal drug Amphotericin B (AmB) which mainly binds with ergosterol, and Aureobasidin A (AbA), a complex sphingolipid biosynthesis inhibitor. Our studies revealed that a non-toxic and low concentration of farnesol can reduce the efficacy of AmB and AbA on yeast cells. This reduction is probably through the alteration in the complex sphingolipid biosynthesis and ATP-binding cassette (ABC) type membrane transport activity. These findings may shed light on a new direction to explore the role of lipid molecules in the antifungal drug resistance mechanisms in pathogenic yeast.

## 1. Introduction

*Candida albicans* is an opportunistic pathogenic yeast that exists as a member of human gut flora (Calderone et al. 2009). Usually, it is a commensal organism, but it can become pathogenic in immunocompromised individuals under a variety of conditions and it causes severe life-threatening infections (Kim and Sudbery 2011). *C. albicans* can grow as both yeast and filamentous formsand can switch between different morphogenic forms, which is highly correlated with its ability to cause disease (Langford et al. 2009). *C. albicans* can secrete the quorum-sensing molecule farnesol which inhibits yeast-to-filamentous form conversion (Nickerson et al. 2006), resulting in mainly actively budding yeasts without any filamentous growth that could affect the drug susceptibility of this pathogen (Hornby et al. 2001). The extensive and long-term usage of antifungal drugs in recent years has caused the rapid development of drug resistance, which leads to major threatto antifungal therapy (Zhai and Lin 2011).

Farnesol is basically a quorum-sensing molecule secreted by *C. albicans* and can inhibit yeast-to-hyphal conversion by regulating the cyclic AMP (cAMP) signalling pathway in *C. albicans* (Uppuluri et al. 2007; Chen et al. 2018). Quorum sensing is a phenomenon where the regulation of gene expression and group behavior is altered in response to changes in cell population density (Miller and Bassler 2001). A previous study reported growth phase-dependent activity of farnesol molecules towards *C. albicans*, and the author reported that minimal cell death was observed at farnesol concentrations up to 300 µM, when starting with stationary-phase inoculum using a defined growth medium (YPD), however, 40 µM farnesol partially inhibited growth, and higher farnesol levels prolonged the lag phase with the inoculum of exponentially growing cells. Moreover, exogenous farnesol levels up to 300 µM do not alter the growth rate; instead, the cells grow as yeasts rather than as filaments (Mosel et al. 2005; Langford et al. 2010). Previous findings suggest the role of oxidative stress due to reactive oxygen species (ROS) generation as a primary cause of farnesol-mediated growth inhibition in the *Saccharomyces cerevisiae* cells moreover, farnesol-mediated ROS generation was not observed in the respiration-deficient petite mutant, which illustrates the role of the mitochondrial electron transport chain as its origin in *S. cerevisiae* cells (Machida and Tanaka 1999). Similarly, another study explored that the Pkc1 signalling pathway regulates farnesol-mediated cell death through the generation of ROS and this is a primary mechanism by which farnesol kills cells (Fairn et al. 2007).

AmB, a commonly used antifungal drug, preferentially binds to ergosterol-containing fungal membranes, (Anderson et al. 2014) used to treat fungal infection caused by pathogenic fungi *C. albicans* and *Cryptococcus neoformans* (Bassetti et al. 2016; Iyer et al. 2021). This binding causes the membrane osmotic integrity to be disrupted, resulting in the leakage of intracellular ions, and inducing fungal cell death (Cavassin et al. 2021). So far, several modes of AmB drug resistance are reported such as the decrease in ergosterol content or mutation in ergosterol biosynthetic pathway genes, which was proposed to be the primary cause responsible for its resistance (Sanglard et al. 2003; Young et al. 2003; Martel et al. 2010). Recently, it has been shown that the deletion of the sphingolipid biosynthetic pathway gene *FEN1* and *SUR4* in *S. cerevisiae*, and its homolog in *C. albicans* sensitise them against polyene AmB (Sharma et al. 2014). Similarly, another study demonstrated that plasma membrane proteolipid 3 protein affects AmB resistance through the sphingolipid biosynthesis pathway (Bari et al. 2015). Moreover, disruption of the interaction between membrane ergosterols and sphingolipids or alteration in membrane lipid composition can modulate the antifungal drug susceptibilities of *C. albicans* (Mukhopadhyay et al. 2002, 2004). In conclusion, these studies highlighted the role of sphingolipid molecules as potential effector in the modulation of antifungal drug susceptibilities.

Yeast *S. cerevisiae* cells can synthesise three types of complex sphingolipids depending on differences in the structure of the polar head group, and the compositions which mainly constitute inositol phosphates (IP) derived moiety namely Inositolphosphoryl-ceramide (IPC), Mannosyl-inositol phosphoryl-ceramides (MIPC), and Mannosyl-di-(inositol phosphoryl)-ceramides (M(IP)_2_C) (Culbertson et al. 1976). AbA is a potent and specific inhibitor of phosphatidylinositol: ceramide phosphoinositol transferase (Aur1p), which catalyzes the first step in complex sphingolipid biosynthesis (Heidler and Radding 1995; Cerantola et al. 2009). Complex sphingolipids are important structural components of the plasma membrane and play an important role in the maintenance of the physical properties of plasma membranes such as membrane fluidity, membrane asymmetry, and acting as signalling molecules (Dickson et al. 2006; Dickson 2008). In this study, we have studied the impact of farnesol on the susceptibility of two drugs, first AmB which mainly targets ergosterol, and secondly, AbA which acts as an inhibitor of complex sphingolipids biosynthesis in *S. cerevisiae* and *Candida spp*. (Sugimoto et al. 2004; Cerantola et al. 2009).

## 2. Material and methods

### 2.1 Strains, media, and growth conditions

*S. cerevisiae* deletion mutants were obtained from the Euroscarf (www.euroscarf.de). Chemicals and yeast growth medium components were obtained from Himedia and Sigma. Stock solutions of AmB (stock 2 mg/ml, Sigma A9528), AbA (stock 2 mg/ml, Takara 630,499), and farnesol (stock 50 mM, Sigma F203) were prepared in DMSO and stored at −20°C until use.

### 2.2 Drug susceptibility by dilution spotting

For drug susceptibility by dilution spotting assays, the strains were grown overnight in synthetic complete (SC) medium, re-inoculated in fresh medium to an OD_595nm_ of 0.1, and grown for 5–6 h at 30°C with shaking at rpm 200 until an OD_595nm_ of 0.6 to 0.8 was reached. The exponential phase cells were harvested, washed, and resuspended in sterile water to an OD_595nm_ of 1.0 (2 X 10^7^ cells/ml). Ten-fold serial dilutions were made in water and 5 μl of each dilution was spotted on desired drug plates. Growth was assessed after the plates were incubated for 2 days at 30°C before taking photographs. The drug minimum inhibitory concentration (MIC) was defined as the concentration at which no growth was observed in the dilution spot assay.

### 2.3 XTT reduction assay

For testing the viability of yeast cells in presence of different concentrations of farnesol, 100 µl of cell suspension (2x10^3^cells/ml in RPMI 1640 medium, pH-7 [supplemented with L-glutamine and buffered with 0.165 M MOPS] from overnight culture in YPD) was added to wells of a microtiter plate. To test farnesol-dependent toxicity the cell suspensions were mixed with 100 µl of two-fold serially diluted farnesol using concentrations of 0 µM, 50 µM, 100 µM, and 200 µM at 37°C for 24 h. At 24 h the metabolic activity of fungal cells was determined by the XTT [2,3-bis(2-methoxy-4-nitro-5-sulfophenyl)-2 H-tetrazolium- 5-carboxanilide sodium salt] reduction assay (Ramage et al. 2001). The XTT tetrazolium salt (Sigma) was dissolved at 0.5 g/litre in phosphate-buffered saline (pH 7.4), filtered sterilised through a 0.2-µm filter, and stored in aliquots at – 80°C. Just before use, an aliquot was thawed and menadione (Sigma; 10 mM prepared in acetone) was added to the XTT solution to a final concentration of 1 µM. One hundred microlitres of XTT-menadione solution were added to yeast cells in microtiter wells and to control wells (for the measurement of background XTT reduction levels); mixed well, and incubated at 37°C in the dark for up to 2 h. A colorimetric change in the XTT reduction (reduced formazan-coloured product formation which is correlated with the metabolic activity of the growing cells) was then measured in a microtiter plate reader (BioTrek Microplate Reader; USA.) at 490 nm. The growth assay in the presence of farnesol was repeated thrice.

### 2.4 Quantitative estimation of ergosterol content

Total intracellular sterols were extracted as reported by Arthington-Skaggs with slight modifications (Arthington-Skaggs et al. 1999). Briefly, 50 ml overnight culture (16 hrs), with or without farnesol (50 µM) treatment, was harvested in pre-weighed 50 ml tubes and washed once with sterile distilled water. The tubes were re-weighed to determine the wet weight of the pellets. Three milliliters of 25% alcoholic potassium hydroxide solution (25 g of KOH and 35 ml of sterile distilled water, brought to 100 ml with 100% ethanol), were added to each pellet and the mix was vortexed for 1 min. Cell suspensions were transferred to 16-mm by 100-mm sterile borosilicate glass screw-cap tubes and were incubated in an 80°C water bath for 2 h. Following incubation, the tubes were cooled at room temperature. Sterols were then extracted with a mixture of sterile distilled water and n-heptane (1:3, v/v) followed by vigorous vortex mixing for 3 min. The heptane layer was transferred to a clean borosilicate glass screw-cap tube and stored at −20°C (not more than 24 h). Before analysis, the aliquot of sterol extract (200 µl of the heptane layer) was diluted in 800 µl of 100% ethanol and was either measured by spectrophotometer at both 281.5 and 230 nm or scanned between 220 and 310 nm. The presence of ergosterol and the late sterol intermediate 24(28) dehydroergosterol [24(28) DHE] in the extracted sample resulted in a characteristic four peaked curve. Ergosterol content was calculated as the percentage of the wet weight of the cell by the following equations:

% ergosterol + % 24(28)DHE = (A281.5/290)×F/ pellet weight,

% 24(28)DHE = (A230/518) × F/ pellet weight,

% ergosterol = (% ergosterol + % 24(28)DHE) − % 24(28)DHE, where F is the factor of dilution in ethanol and 290 and 518 are the E values (in percentage per cm) determined for crystalline ergosterol and 24(28)DHE, respectively. The experiments were carried out thrice in duplicates.

### 2.5 Thin layer chromatography

Lipids were extracted from yeast strains *C. albicans* and *S. cerevisiae* as described previously (Toume and Tani 2016) with some minor modifications. Briefly, 3 OD cells were extracted using 350 *μ*l of extraction liquid ethanol/water/diethyl ether/pyridine/15 M ammonia in the v/v ratio (15:15:5:1:0.018) by incubation at 65°C for 15 mins. Then the extract of lipids were centrifuged at 10,000 g for 1 min and then extracted once more in the same manner. The resulting supernatants were dried and subjected to mild alkaline treatment using mono-methylamine. It was done by dissolving the lipids in 130 *μ*l of 40% mono-methylamine/ water in the v/v ratio of 10:3 and incubating at 53°C for one hour. The solutions were dried and the resulting lipids were dissolved in 50 *μ*l of chloroform/methanol/water in a v/v ratio of 5:4:1. The lipids were loaded by TLC spotting capillary tubes and separated in a Merck TLC silica gel plate with chloroform/methanol/4.2 M ammonia in a v/v ratio of 9:7:2. Then the plates were visualised using UV light.

### 2.6 RNA isolation and qRT‑PCR

Total RNA from yeast cells was extracted using GeneJET RNA Purification Kit (K0732) according to the manufacturer’s protocol. 500 ng total RNA was used to obtain cDNA according to the protocol of the Thermo RevertAid First Strand cDNA Synthesis Kit (K1621). The qRT-PCR analysis was performed in a volume of 10 μL using PowerUp™ SYBR™ Green Master Mix (A25741-Applied Biosystems™), and 5X diluted cDNA as the template. *ACT1* was used as the internal reference gene. The specificity of the primers was confirmed by melting curve analysis. The generated Ct values of the target genes were normalised using the reference gene *ACT1*. Relative expression was calculated using the 2^−ΔΔ^Ct method and expressed as a fold change with respect to the control (Livak and Schmittgen 2001).

### 2.7 Cellular localisation of Pdr5p in *S.* *cerevisiae*

To localise ScPdr5p indirect immunofluorescence was performed as per the published protocol (Severance et al. 2004). Briefly, exponentially growing cultures were treated with 50 µM farnesol and incubated for 2 hrs. 1 ml culture was harvested and fixed in 4% paraformaldehyde for 2 hrs incubation at 30°C. Then fixed cells were washed with 100 mM PBS buffer and resuspended in 1.2 M sorbitol buffer. Spheroplasting was done by addition of 10 µl β-mercapto-ethanol and 10 µl of 10 mg/ml zymolase and further incubated at 30°C for minutes. After washing 15 µl spheroplast adhered to a poly-lysine coated cover slip and permeabilized by treatment with 0.4% Triton X-100 in PBS for 2 min. This was followed by 30 min block in 1% BSA and overnight incubation with mouse monoclonal anti-HA primary antibody (Invitrogen-326700) (1:200 dilution) at 4°C. Further incubation after washing with secondary antibody Alexa Fluor 488 conjugated goat anti-mouse antibody (Invitrogen-11,001) (1:500 dilution) was done for 4 hrs at room temperature. Images were obtained with an inverted LSM510 META laser scanning confocal microscope using 100 X objectives and 488 nm laser.

## 3. Results

### 3.1 Effect of farnesol on the growth of yeast cells

**Figure 1.**
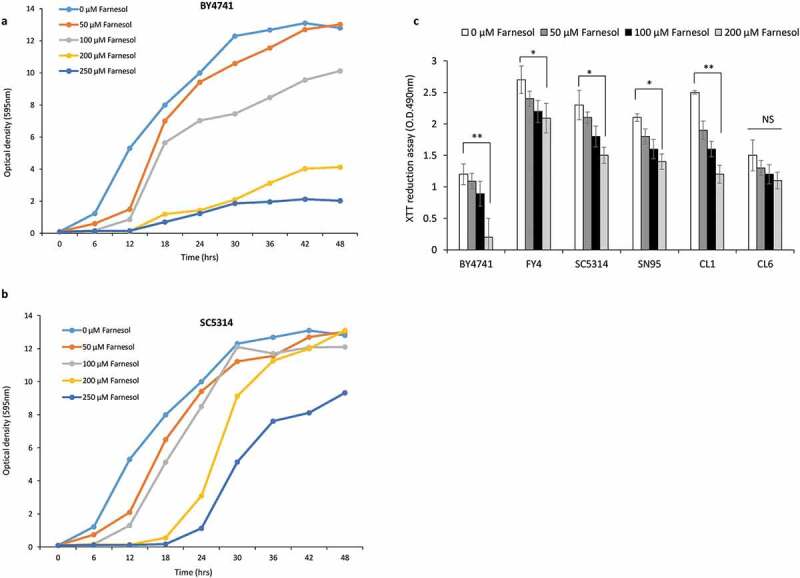
Growth curve analysis for the **(a)** *S. cerevisiae* (BY4741) and **(b)** *C. albicans* (SC5314) in presence of a different concentration of farnesol. Experiments were repeated thrice with comparable results. The values shown are the average of two replicates from one representative experiment. **(c)** XTT reduction assay for *Candida* and *S.cerevisiae* strains in presence of different concentrations of farnesol. Error bars represent the standard error of the mean (SEM) of three individual experiments; using two biological replicates for each sample (Student’s t-test at p < 0.05). Asterisks (*) onto the bars show significant differences.

In this study, we assessed the impact of farnesol exposure on the growth of *S. cerevisiae* (BY4741 and FY4), *C. albicans* (SC5314 and SN95), and clinical isolates of *C. lusitaniae* (CL1 and CL6) strains. To evaluate the effect of farnesol on the growth of yeast cultures, 1 × 10^6^ cells/ml of BY4741 and SC5314 cells were treated with different concentrations of farnesol *i.e*. 0, 50, 100, 200, and 250 μM, and growth was assessed by optical density (595 nm) using a spectrophotometer (Figure 1(a and b)). Notably, cell growth was impaired in liquid culture at a high concentration of farnesol *i.e*. 250 μM in both the strains, more severely affecting BY4741. Farnesol concentrations of 50 μM and 100 μM did not have a significant inhibitory effect, though there was a decrease in exponential growth rate as compared to untreated control. Moreover, there was an increase in the lag phase of 6 hrs in the case of 50 μM and 100 μM farnesol for SC5314 and BY4741. Results show that farnesol affects the exponential growth rate but does not inhibit growth, though the possibility of compromised cell viability cannot be ruled out. To further confirm these results, we included *C. albicans* (SN95), C. *lusitaniae* (CL1 and CL6), and *S. cerevisiae* (FY4) and compared the growth in presence of different concentrations (0 μM, 50 μM, 100 μM, and 200 μM) of farnesol using an XTT ((2,3-Bis-(2-Methoxy-4-Nitro-5-Sulfophenyl)-2 H-Tetrazolium-5-Carboxanilide) reduction assay to quantify the growth. The growth rate of all the strains differed to a different extent for all concentrations of farnesol used (Figure 1c). For *S. cerevisiae* BY4741 strain, a significant difference in growth between control and 250 µM farnesol treatment was noted when incubated for 24 hrs, however, no significant difference in growth was observed between control and cells treated with 50 μM and 100 μM farnesol. For other strains also there was no significant decrease in growth in presence of different concentrations of farnesol. Thus, our data indicated an increase in lag phase with increasing farnesol concentration and a similar growth rate at lower farnesol concentration as compared to control for certain strains.

### 3.2 Farnesol supports the growth of yeast against AmB drug

**Figure 2.**
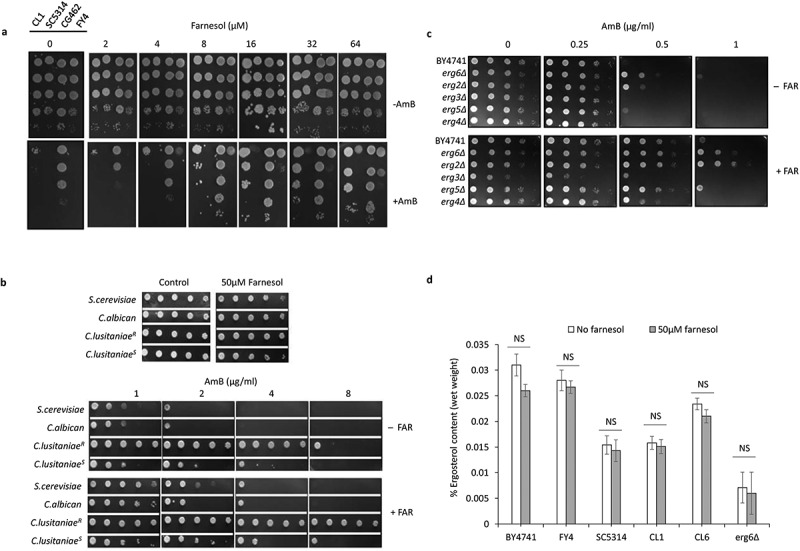
**(a)** Optimising farnesol concentration which modulates AmB (1 µg/ml) drug susceptibilities in strains of *S.* *cerevisiae* (FY4) and *C.* *albicans* (SC5314), *C.* *glabrata* (CG462), *C. lusitanie* (CL-1) **(b)** 50 µM farnesol (+FAR) sufficient to support growth against AmB in strains of *S.* *cerevisiae* and *Candida* **(c)** Modulation of AmB drug susceptibility of ergosterol biosynthetic deletion mutants of *S.* *cerevisiae* in presence of 50 µM farnesol (+FAR), No farnesol (-FAR). **(d)** Estimation of ergosterol content with and without farnesol (50 µM). Values are expressed as a percentage of the wet weight of the cell pellets. Error bars represent the standard error of the mean (SEM) of three individual experiments; using two biological replicates for each sample (Student’s t-test at p < 0.05). NS onto the bars shows no significant differences.

According to the earlier reports, it is expected that in *C. albicans* different concentrations of farnesol trigger different effects on cellular morphology (Hornby et al. 2001; Ramage et al. 2002). Apart from this, some effects of farnesol required a much higher concentration of farnesol than that needed to block germ tube formation in presence of serum (Nickerson et al. 2006). Therefore, to identify the minimal levels of farnesol needed to effectively modulate the AmB resistance, a serial dilution spotting assay was done for strains CL1, SC5314, CG462, and FY4 using different concentrations (1–64 µM) of farnesol, with or without 1 µg/ml AmB. Our results indicate that the maximum effect of farnesol on AmB resistance (increased MIC) was seen at the highest farnesol concentrations (64 µM) for all strains except *C. glabrata* where no effect of farnesol on AmB resistance was observed (Figure 2a). Hence, a minimum concentration of 32 µM to 64 µM farnesol is sufficient for the growth of strains CL1, SC5314, and FY4 in the presence of 1 µg/ml AmB, thereby indicating that much higher levels of farnesol are needed for increasing AmB resistance as compared to 2–4 µM of farnesol produced by stationary phase cultures of *C. albicans*. In addition to the above, farnesol-mediated AmB susceptibility was also analysed using *C. lusitaniae* AmB sensitive strain (CL1), *C. lusitaniae* AmB resistant strain (CL6), *C. albicans* strain (SC5314), and *S. cerevisiae* strain (FY4) and serial dilution of yeast culture were spotted onto plates containing different concentration of AmB (1–8 µg/ml), with or without 50 µM farnesol (Figure 2b). Interestingly, we observed that all the strains showed increased resistance to AmB in presence of farnesol, although to a different extent compared to the control (AmB without farnesol).

The growth support effect was seen in *C. lusitaniae* AmB sensitive strain CL1 in which MIC of AmB increased from 2–4 µg/ml (without farnesol) to 8–16 µg/ml (with farnesol) demonstrating a 2-4-fold increase in MIC, similarly resistant strain CL6 in absence of farnesol MIC of 8–16 µg/ml showed growth well above its MIC in presence of farnesol in dilution spot assay. In addition, *C. albicans* (SC5314) and *S. cerevisiae* (FY4) strains also exhibited AmB resistance about 2-4-fold above their MIC of 1–2 µg/ml in presence of farnesol, in dilution spot assay. Differences in AmB MIC of strains in dilution spotting assay could be attributed to the difference in stock preparation, handling of the drug, and the number of cells present, however, we always compare the results with wild-type control strains on the same plate. In conclusion, the potential role of farnesol in increasing the AmB resistance of all the strains tested except for *C. glabrata* where we did not observe any remarkable changes in AmB MIC, with or without farnesol.

Since the resistance to AmB mainly involves alteration in ergosterol type or content (Young et al. 2003), we rule out this possibility, by analysing the AmB sensitivity of ergosterol biosynthetic deletion mutant of *S. cerevisiae* in the presence of farnesol, which suggests farnesol supports the growth of all the tested strains (Figure 2c). In addition, we also measured the ergosterol content in *S. cerevisiae* and *Candida* strains with and without farnesol, however, the change in ergosterol content with farnesol was not significantly different from the control strains (Figure 2d). Together, these results show that the effect of farnesol in decreasing the susceptibility of AmB is not mediated through, the ergosterol biosynthetic pathway, and the AmB resistance is not always associated with a reduction in ergosterol content.

### 3.3 Farnesol reduces the effect of AbA by altering the complex sphingolipid content

**Figure 3.**
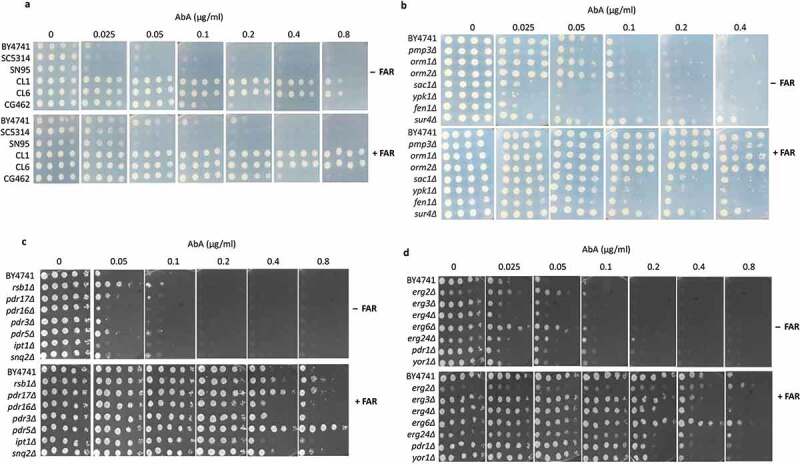
Growth assay by dilution spot assay **(a)** for analysing farnesol-mediated modulation of AbA drug resistance in strains of *S. cerevisiae* and *Candida* spp. **(b)** sphingolipid biosynthetic and regulatory genes of *S. cerevisiae* **(c)** pleiotropic drug response genes of *S. cerevisiae* involved in membrane transport **(d)** ergosterol biosynthesis deletants of *S. cerevisiae*. Ten-fold serial dilutions of cells were spotted onto synthetic complete agar plates with the indicated concentration of AbA and farnesol (50 µM). Plates were incubated at 30°C for 2 days before being photographed. 50 µM farnesol (+FAR), No farnesol (-FAR).

The complex sphingolipids, are important structural components of the plasma membrane and play an important role in maintaining plasma membrane asymmetry, dynamics, and signalling (Gault et al. 2010; Gururaj et al. 2013). In yeast complex sphingolipids contain inositol phosphate derived from phosphatidylinositol lipids. Yeast synthesises three complex sphingolipids namely IPC, MIPC, and M(IP)_2_C (Dickson 2008). AbA is a potent and specific inhibitor of Phosphatidylinositol: ceramide phosphoinositol transferase (Aur1p), which catalyses the first step in complex sphingolipid synthesis (Cerantola et al. 2009). While investigating the role of the sphingolipid in antifungal drug resistance, we hypothesised that farnesol might be responsible for the increased resistance through sphingolipid biosynthesis. Therefore, the impact of farnesol on AbA, a complex sphingolipid biosynthesis pathway inhibitor was analysed using *S. cerevisiae* and *Candida* strains. Interestingly, we observed the MIC of the AbA drug in *S.cerevisiae* strain (BY4741), *C. albicans* (SC5314, SN95), *C. glabrata* (CG462), and *C. lusitania* (CL1 & CL6), were increased in the presence of 50 µM farnesol by 2 to 4-folds (Figure 3a) which suggests that farnesol is one of the factors that influence AbA drug susceptibility. Since farnesol enhances the drug tolerance of all the tested strains of *S. cerevisiae* by about 2 to 4-folds, it appears that it acts through the sphingolipid pathway. To further explore the role of farnesol-mediated AbA resistance, first, we screened selected gene deletion mutants of *S. cerevisiae* that are directly involved in the regulation of sphingolipid biosynthesis and its homeostasis (Figure 3b). In addition, we have also screened the selected gene deletion mutants of *S. cerevisiae* which encode for drug efflux pump or are involved in pleiotropic drug response that can also affect our farnesol-mediated AbA drug susceptibility phenotype. Interestingly, we have identified a pleiotropic drug response 5 *(PDR5)* gene encoding plasma membrane ATP-binding cassette (ABC) transporter, which in the presence of farnesol affects the AbA resistance substantially out of all the strains tested as compared to the parent strain (Figure 3c). These data demonstrate that *PDR5* is crucial for farnesol-mediated increased AbA resistance, while other genes less influence AbA resistance. Previous studies on ATP-binding cassette transporters in *S. cerevisiae* suggested that *PDR5* controls the asymmetric distribution of phospholipids and regulates the permeability of AbA across the plasma membrane, this conclusion supports our plate-based screen data where *pdr5* deletant is more resistant to AbA, which was further enhanced upon farnesol treatment.

**Figure 4.**
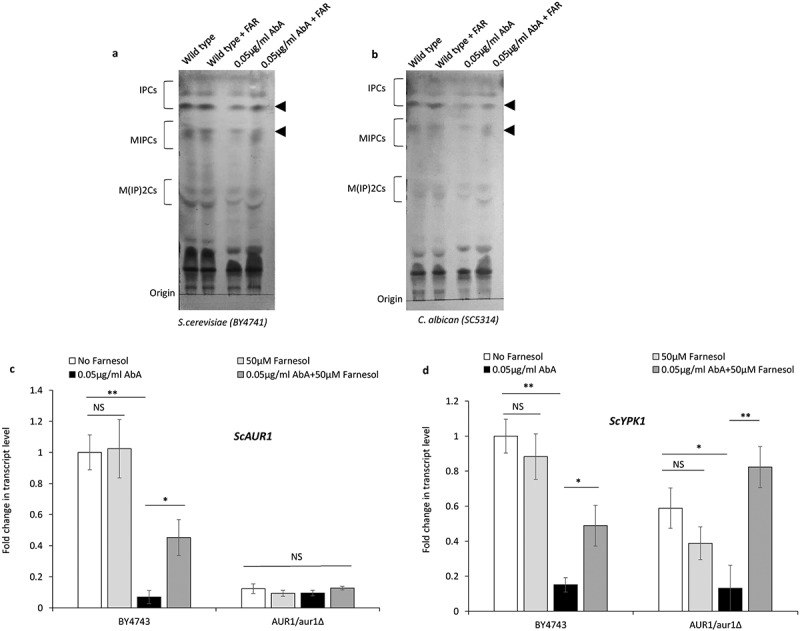
Qualitative analysis of complex sphingolipids using thin layer chromatography in presence of 0.05 µg/ml AbA with and without 50 µM farnesol (+FAR) in **(a)** *S. cerevisiae* (BY4741) and **(b)** *C. albicans* (SC5314), a black triangle denote alteration in IPCs and MIPCs contents. **(c and d)** Quantitative real-time PCR for transcript‑level analysis of *ScAUR1* and *ScYPK1* in presence of 0.05 µg/ml AbA, with and without 50 µM farnesol (+FAR) in wild type (BY4741) and *AUR1/aur1∆* heterozygous strain of *S. cerevisiae*. The expression level of the transcript is displayed after normalisation to *ScACT1*. Error bars represent the standard error of the mean (SEM) of three individual experiments; using two biological replicates for each sample (Student’s t-test at p < 0.05). Asterisks (*) onto the bars show significant differences. NS onto the bars shows no significant differences.

Sphingolipids and ergosterol act as major yeast plasma membrane components, and interaction between these molecules might play a crucial role in drug susceptibility (Mukhopadhyay et al. 2004), hence to rule out the possible involvement of ergosterol in AbA drug susceptibility, we also analyse the AbA susceptibility of the ergosterol biosynthetic pathway mutant in presence of farnesol, however, we did not find any correlation between ergosterol biosynthesis and AbA drug resistance (Figure 3d). From these data, we concluded that sphingolipids rather than ergosterol molecules are a major player in the modulation of AbA drug resistance. To explore further in more detail, we analysed the complex sphingolipid content using thin-layer chromatography with and without farnesol in presence of AbA in *S. cerevisiae* (BY4741) and *C. albicans* (SC5314). Our results demonstrated that the presence of farnesol does not influence complex sphingolipid contents as compared to control strains, however, an increase in the band intensity of the complex sphingolipid contents IPCs and MIPCs in presence of farnesol was observed only upon the addition of AbA. These data suggest that farnesol reduces the effect of AbA by simply reducing the effective dose of AbA which leads to less inhibition of complex sphingolipid biosynthesis (Figure 4(a and b)). These results possibly also explain a correlation between *PDR5* deletant resistance against AbA with and without farnesol on the dilution spot assay. To further explore the details, we analysed the expression of the *AUR1* gene in *S. cerevisiae* which is a direct target of AbA (Hashida-Okado et al. 1996). Since the *AUR1* gene is essential for survival, so we selected *AUR1* heterozygous deletion mutants (*AUR1/aur1∆)* as a negative control for transcript analysis. Consistent with the above results we observed that in wild-type cells (BY4741) farnesol lead to an increase in the transcript levels of IPC synthase gene *AUR1* only in presence of AbA, however, farnesol does not affect *AUR1* transcript level alone, moreover another complex sphingolipid biosynthesis regulator Serine/Threonine protein kinase; *YPK1* gene (Muir et al. 2014) of *S. cerevisiae* which greatly influences complex sphingolipid biosynthesis also shown to increase in the transcript level in presence of farnesol with AbA (Figures 4(c and d)). These data demonstrate that possibly reduced AbA uptake inside cells upon farnesol treatment leads to growth support of yeast cells against AbA.

### 3.4 ABC-type transporters induced upon farnesol treatment

**Figure 5.**
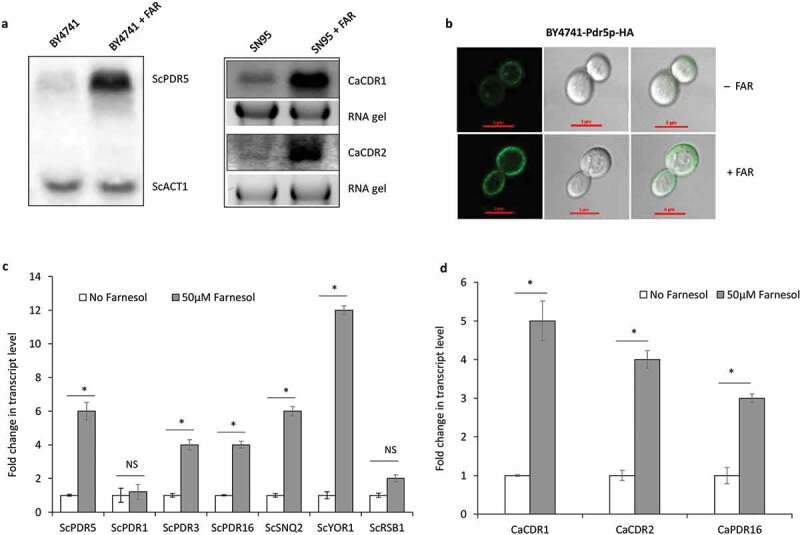
**(a)** Northern blot hybridisation with indicated probes for the expression of *S. cerevisiae* (BY4741) *PDR5* and *C. albicans* (SN95) *CDR1* and *CDR2* in presence of 50 µM farnesol (+FAR). Total RNA (20 µg) was loaded per lane for northern blot hybridisation analysis and the 18S rRNA region of the ethidium bromide-stained gel is shown as a loading control, Adobe Photoshop software was used to process images. Northern blots have been cropped to show specific bands of interest, but are from the same membrane for each boxed figure **(b)** Intense localisation of *S. cerevisiae* (BY4741) Pdr5p-HA on plasma membrane upon 50 µM farnesol treatment, scale bar 5 µm on each image **(c)** Transcript‑level analysis of different *ScPDRs* of *S. cerevisiae* in presence of 50 µM farnesol (+FAR). The expression level of the transcript is displayed after normalisation to *ScIPP1*. Error bars represent the standard error of the mean (SEM) of three individual experiments; using two biological replicates for each sample (Student’s t-test at p < 0.05). Asterisks (*) onto the bars show significant differences. NS onto the bars shows no significant differences. **(d)** Transcript‑level analysis of *CaCDR1, 2* and *CaPDR16* of *Candida* in presence of 50 µM farnesol (+FAR). The expression level of the transcript is displayed after normalisation to *CaACT1*. Error bars represent the standard error of the mean (SEM) of three individual experiments; using two biological replicates for each sample (Student’s t-test at p < 0.05). Asterisks (*) onto the bars show significant differences.

ATP-binding cassettes (ABC) type transporters and Major Facilitators Superfamily (MFS) transporters have been implicated in the development of multi-drug resistance phenotypes in various pathogenic eukaryotes and yeasts (Sipos and Kuchler 2006; Buechel and Pinkett 2020). Multidrug resistance phenotype in yeast is known as pleiotropic drug resistance (PDR) and is caused by the increased expression of genes that encode these non-specific drug-efflux pumps belonging to the ABC or MFS family of transporter proteins (Cannon et al. 2009). To rule out the possible role of these drug transporter, we analysed the expression of *PDR5* in *S. cerevisiae* and *CDR1* and *CDR2* in *C. albicans* (Pourakbari et al. 2017) using northern blot, and data suggest that these transporters get overexpressed upon farnesol treatment (Figure 5a). Moreover, the membrane localisation of HA-tagged Sc*Pdr5p* was more intense upon farnesol treatment (Figure 5b). Furthermore in-depth analysis of the transcription of several ABC-type transporters in *S. cerevisiae* and *C. albicans* suggests that there is a global change in the expression of several transporters in presence of farnesol (Figure 5(c and d)). The role of these genes in AbA tolerance was well established from a previous study which reported that overexpression of the *PDR16* gene confers AbA resistance (Katsuki et al. 2018), moreover mutant strains lacking the ABC transporter *PDR5* and *YOR1* were found to exhibit differential resistance to AbA (Khakhina et al. 2015) and similarly in our study, we also found the consistent role of *PDR5* in AbA drug resistance modulation which upon deletion further enhances the farnesol-mediated AbA tolerance. In conclusion, we have shown that possibly *PDR* type transporter act as an important regulator of AbA drug resistance. These data suggest that the existence of a *PDR* type transporter is necessary for farnesol-mediated AbA resistance modulation.

## 4. Discussion

The major focus of this part of the study was directed towards investigating the effect of farnesol on AmB sensitivity. AmB drug susceptibility testing in presence of farnesol using *Candida* and *S. cerevisiae* strains revealed that the presence of 50 µM farnesol modulated the AmB sensitivity about 2-4-fold, indicating a potential role of farnesol in increasing the AmB resistance. Our study also indicates that a minimum concentration of 32 µM to 64 µM farnesol was sufficient for decreasing the AmB susceptibility for all the tested strains of *Candida* and *S. cerevisiae*. Farnesol is increasingly produced with the age of the culture at a reported estimated concentration of 10–50 μM (Weber et al. 2010). Hence, the concentration at which we observed the reduction in AmB susceptibility was well within the physiologically range produced by *C. albicans* but much higher than that needed to block 50% of germ tube formation in N-acetyl glucosamine stimulated assay *i.e*. 1.2 µM of farnesol (Mosel et al. 2005). In this study, we have uncovered a novel role of farnesol in the drug resistance of AmB and AbA. Several studies have reported AmB molecule mainly targets ergosterol and alteration in ergosterol type or content modulates AmB resistance in *C. lusitaniae, C. albicans, C. glabrata* and *S. cerevisiae* (Young et al. 2003; Hull et al. 2012). Since ergosterol molecules interact physically as well as functionally with sphingolipids, and the biosynthesis of sphingolipids is closely coordinated with that of sterols (Gulati et al. 2010; Hannich et al. 2011), hence sphingolipids molecule could also modulate AmB resistance. To conclude, here we report a novel observation that isoprenoid farnesol plays an important role in modulating AmB resistance in yeast species and the phenomenon is specific. Moreover, to explore the possible mechanism behind this effect, it is observed that this phenomenon is independent of the already known mechanisms of AmB resistance. This observation further strengthens our data that farnesol can modulate the activity of other antifungal drug resistance. Our results reveal that sphingolipid molecules play an important role in AmB resistance mechanism and hence in-depth study is warranted to unravel the molecular basis of this modulation in AmB susceptibility and the mechanism/s involved add more knowledge to the known mechanism of AmB resistance.

Furthermore, the farnesol-mediated increase in resistance to AbA provides evidence that farnesol likely modulates the sphingolipid biosynthetic pathway, since the presence of farnesol supports complex sphingolipid biosynthesis in the presence of AbA. Recently a study indicated that *PDR16*, a member of phosphatidylinositol transfer proteins and its paralog *PDR17*, acts as multicopy suppressor genes that confer resistance to AbA, however, *PDR16* lipid binding defective mutant does not provide resistance against AbA in *S. cerevisiae* (Katsuki et al. 2018), thus, it is possible that the acquisition of AbA resistance in *pdr5∆* cells is partly involved *PDR16* which further enhanced upon farnesol treatment. In another study, it was reported that strains defective in *PDR5* and *YOR1* show reduced susceptibility to AbA and this phenotype likely involves altered activity of plasma membrane-localised flippase complex Dnf1/Lem3 and Dnf2/Lem3, which regulates the permeability/efflux of AbA by controlling the unknown AbA exporter present in the plasma membrane (Khakhina et al. 2015). In addition, the function of Lem3 was already shown to be required for myriocin (sphingolipid biosynthetic pathway inhibitor) drug uptake by directly measuring the intracellular myriocin level (Yamane-Sando et al. 2014). ABC transporter encodes drug-efflux pumps and implicates the drug resistance but these transporters can also control the activity of other plasma membrane proteins such as defective internalisation of high-affinity tryptophan permease Tat2 upon *PDR5* or *YOR1* deletion (Johnson et al. 2010). Pdr5 possibly regulates the activity of Lem3, a flippase of the plasma membrane which might affect AbA drug susceptibility as discovered by (Khakhina et al. 2015), however, *PDR5* does not involve in the direct efflux of AbA drug. The conclusion from these studies provides a possible explanation that loss of *PDR5* inhibits the uptake of AbA or altered sphingolipid distribution, which was further enhanced upon farnesol treatment. These data provide additional insight into the importance of ABC-type transporter in influencing lipid distribution and regulating membrane transporters activity that might involve in AbA transport. To explain our results, we propose that possibly farnesol competes with AbA in its interaction with *AUR1* or farnesol alters the plasma membrane sphingolipids content that affects AbA interactions with membrane leading to the altered ability of the drug to enter the cell or farnesol forms micelles external to the cell and the lipophilic drug AbA sequesters itself in those micelles, effectively lowering the AbA concentration. Another possibility is that farnesol positively regulates a membrane protein which could selectively efflux out the AbA from the cells. Further detailed investigation of the effects of farnesol on the action of AmB and AbA will provide new insights into the modulation of antifungal drug susceptibility in pathogenic yeast. Efforts are underway to elucidate the exact mechanism of how farnesol confers the AmB and AbA drug tolerance.

## Data Availability

The datasets used and/or analyzed during the current study are available from the corresponding author upon reasonable request.
